# A radiomic model for noninvasive prediction of PD-L1 and VETC expression in hepatocellular carcinoma using enhanced abdominal CT

**DOI:** 10.3389/fonc.2025.1696376

**Published:** 2025-12-12

**Authors:** Weidong Wang, Junrong Lu, Yongcong Yan, Kai Wen, Gefan Guo, Zhenyu Zhou, Zhiyu Xiao

**Affiliations:** Sun Yat-sen Memorial Hospital, Sun Yat-sen University, Guangzhou, China

**Keywords:** hepatocellular carcinoma, PD-L1, VETC, radiomics, machine learning, random forest

## Abstract

**Background:**

Hepatocellular carcinoma (HCC) is a prevalent malignant tumor that is associated with significant morbidity and mortality. Programmed cell death 1 ligand 1 (PD-L1) and Vessel Encapsulating Tumor Clusters (VETC) are critical biomarkers influencing immune evasion and metastasis, making them pivotal for guiding treatment decisions. However, it is challenging to obtain pathological samples from some patients because of factors such as advanced tumor stage and poor liver function.

**Objective:**

This study aimed to develop an AI-based imaging model to non-invasively predict PD-L1 and VETC expression in HCC patients, addressing the challenge of limited histopathological data.

**Methods:**

This retrospective study included 162 HCC patients diagnosed between January 2017 and December 2022. Patients were randomly divided into training and test sets (8:2). Radiomic features were extracted from CT images, and various machine learning algorithms were used to construct predictive models and assess their accuracy in predicting PD-L1 and VETC expression.

**Results:**

A total of 2,286 features were extracted from the enhanced abdominal CT images. Among them, seven features were associated with PD-L1 expression and 10 with VETC expression. The Random Forest (RF) model demonstrated good calibration and fit, emerging as the most effective with an AUC of 0.834 (95% CI: 0.752–0.915) for PD-L1 and 0.883 (95% CI: 0.818–0.949) for VETC in the training set, while achieving AUCs of 0.740 (95% CI: 0.541–0.939) for PD-L1 and 0.705 (95% CI: 0.488–0.922) for VETC in the test set.

**Conclusion:**

The radiomics model derived from enhanced abdominal CT demonstrates its potential as a noninvasive tool for predicting the expression of PD-L1 and VETC in HCC patients.

## Introduction

Hepatocellular carcinoma (HCC) is a highly prevalent malignant tumor of the digestive system, characterized by elevated morbidity and mortality rates (1, 2). Surgical resection is often not feasible upon diagnosis, resulting in poor prognosis.

Within the cancer immune cycle, the immune checkpoint PD-1 and its ligand PD-L1 play pivotal roles in tumor evasion of immune-mediated apoptosis, suppression of T- and B-cell activation, and facilitation of tumor metastasis and progression. Elevated PD-L1 expression correlates with these phenomena (3–5). Targeted therapy for the PD-1/PD-L1 axis effectively restores immune cell functionality and suppresses tumor growth, indicating that PD-L1 expression is a potential biomarker for guiding subsequent treatment strategies.

Vessel encapsulating tumor clusters (VETC) are tumor lesions surrounded by capillary endothelial cells that facilitate hematogenous metastasis, resulting in an unfavorable prognosis after radical surgical resection. Consequently, VETC serves as a robust adverse-prognostic indicator (6–8). Meanwhile, VETC guides in selecting subsequent treatment strategies, such as sorafenib, which demonstrates a notable increase in overall survival for VETC-positive patients but does not extend survival in VETC-negative patients (9).

A definitive pathological diagnosis is not currently required for HCC diagnosis. Many patients who are ineligible for surgical resection and decline biopsy procedures lack the pathological evidence to guide subsequent treatment decisions. Importantly, the assessment of PD-L1 and VETC expression requires histopathological evaluation. Therefore, noninvasive and convenient method for determining PD-L1 and VETC expression in patients without histopathological data is needed. Artificial intelligence (AI), specifically radiomics technologies applied to medical imaging, supports the need for noninvasive and precise medical interventions. AI has already shown promise in analyzing the cytoplasmic matrix and microenvironment of liver cancer cells, distinguishing between liver cancer types, and predicting therapeutic responses to refine treatment strategies (10). AI-driven CT image analysis has the potential to predict PD-L1 and VETC expression, thereby informing the selection of follow-up treatment for HCC. Therefore, this study aimed to develop a radiomics model using enhanced abdominal CT to non-invasively predict PD-L1 and VETC expression levels in HCC patients, thereby providing treatment decision support for patients who are unable to obtain pathological samples.

## Materials and methods

In this study, we reviewed patients diagnosed with HCC at our institution between January 2017 and December 2022 who underwent abdominal CT enhancement scans and pathological examinations after radical surgical resection. The hospital’s ethics committee approved this study and waived the requirement for informed consent. After screening, 162 patients were enrolled in the study.

The inclusion criteria were as follows: 1) histologically confirmed HCC with available PD-L1 and VETC expression assessment via histopathology after resection; 2) complete abdominal triple-phase imaging data available, with an interval of less than 2 weeks between pathology and imaging examinations; 3) treatment-naive status (no prior surgical, interventional, or systemic treatment) before surgical resection and imaging examination; and 4) complete clinical and laboratory data available.

Exclusion criteria included: 1) Suboptimal imaging quality due to significant motion artifacts; 2) Coexistence of other primary malignancies; 3) Incomplete clinical pathological data for comprehensive analysis.

**Figure 1:**
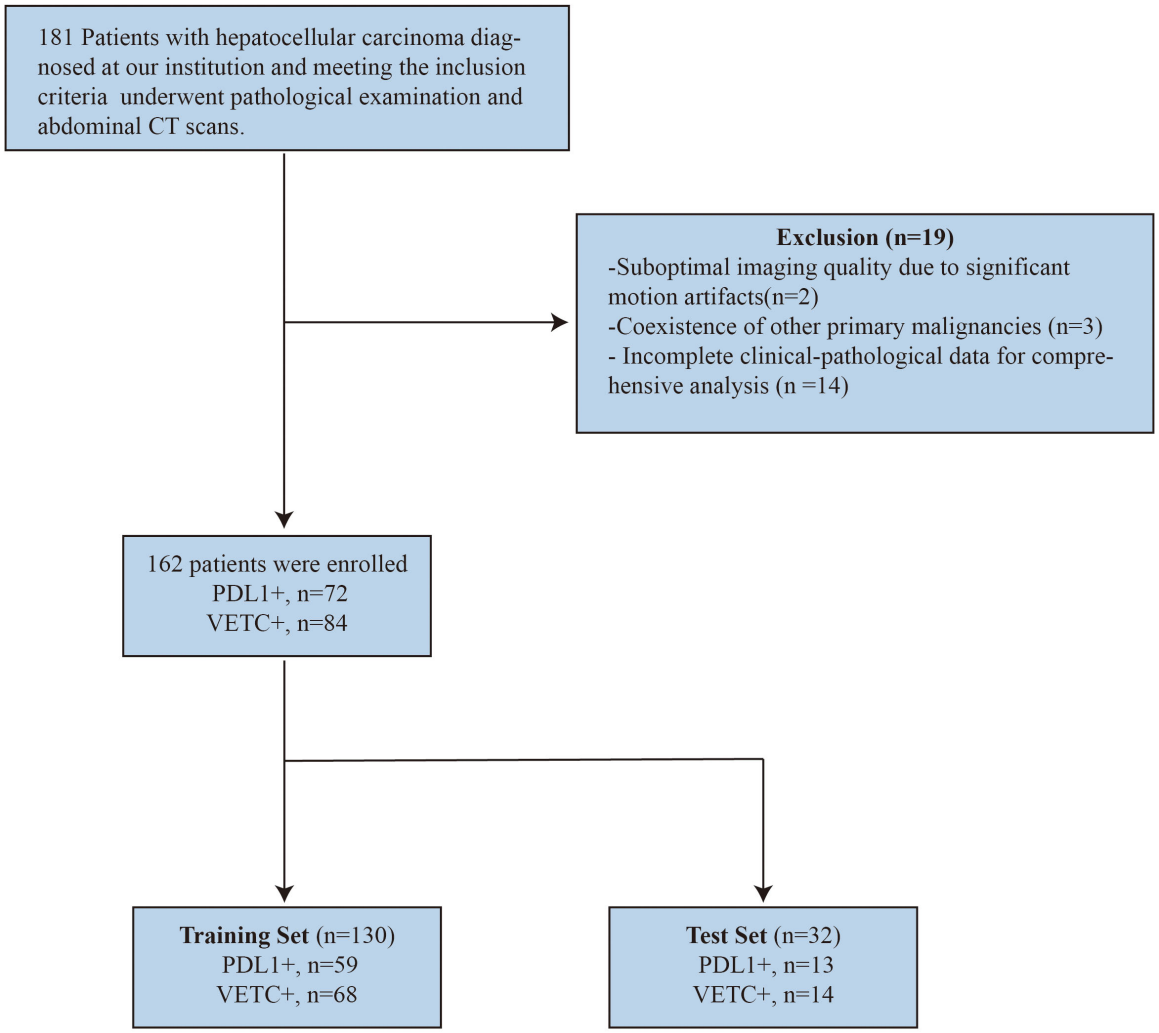
Flowchart of patient enrollment and study design.

To ensure an appropriate sample distribution, the dataset was randomly divided into training and test sets using an 8:2 ratio. The screening and grouping processes are illustrated in **Figure 1**.

### Assessment of PD-L1 and VETC expression

The PD-L1 expression and VETC extent were independently evaluated by two experienced pathologists. In cases of disagreement, a third pathologist was consulted to discuss and reach a consensus. All three pathologists were blinded to the patients’ clinical, laboratory, and imaging findings before reaching a final decision.

VETC is defined as the occurrence of sinusoid-like vessels that form web-like networks and encapsulate individual tumor clusters, either partially or entirely within the tumor, as observed in CD34 immunostaining. For CD34 immunostaining, the tissue sections were deparaffinized, rehydrated, and subjected to antigen retrieval using citrate buffer (pH 6.0). The primary antibody, CD34 QBEnd/10 (Quanhui International, China), was applied at a dilution of 1:100, followed by incubation with a horseradish peroxidase (HRP)-conjugated secondary antibody. Diaminobenzidine (DAB) was used for chromogenic detection, and hematoxylin counterstaining was performed on the sections. The extent of VETC was semi-quantitatively assessed in increments of 5% from 0% to 100%. A VETC index of 5% or higher classifies the tumor as VETC positive (VETC+), whereas below this threshold, it is classified as VETC negative (VETC−) (11).

For PD-L1, the intensity of staining was rated on a scale from 0 (negative) to 3 (dark brown). Immunostaining for PD-L1 was performed using the PD-L1 22C3 pharmDx assay (Dako, Agilent Technologies, USA). Tissue sections were incubated with the primary antibody PD-L1 22C3 (clone 22C3, prediluted) after heat-induced epitope retrieval. Detection was achieved using the EnVision FLEX+ visualization system (Dako), followed by DAB chromogen and hematoxylin counterstaining. The proportion of positively stained cells was scored as follows: 0% (0 points), 1%–25% (1 point), 26%–50% (2 points), 51%–75% (3 points), and 76%–100% (4 points). The total IHC staining score was calculated by multiplying the intensity score by the proportion score (12, 13). A total score exceeding 4 points indicated high expression (PD-L1+), whereas a score of 4 or less indicated low expression (PD-L1−).

### CT acquisition

Patients underwent contrast-enhanced liver CT within 2 weeks before treatment initiation. CT scans were performed in the axial plane with 1.25 mm-thick sections using a 64-row multi-detector row CT scanner (Discovery CT750hd; GE Healthcare, Waukesha, Wis, USA). The liver protocol includes non-contrast CT followed by a subsequent contrast-enhanced scan encompassing the arterial, portal, and delayed phases. Scanning to capture contrast-enhanced images was conducted at 25 s, 65 s, and 180 s after the injection of ioversol (injection rate of 3 mL/s, dose of 1.0 mL/kg of body weight). The scan parameters were as follows: tube current, 250 mA–350 mA; tube voltage, 120 kVp; rotation time of the tube, 0.8 s; pitch, 1.375:1; slice thickness, 1.25 mm; and interval thickness, 1.25 mm.

### Image segmentation and radiomic feature extraction

Two blinded radiologists manually delineated the ROIs on arterial phase CT images using the uAI Research Portal (United Imaging Intelligence). The arterial phase was specifically selected because HCC typically exhibits intense hyperenhancement during this phase, providing superior tumor-to-liver contrast and texture details compared to other phases. This phase is critical for capturing the intratumoral heterogeneity associated with microvascular proliferation (VETC) and immune expression. Consequently, slice-by-slice tracing was performed to generate volumes of interest (VOIs), excluding peritumoral zones, major vessels (>3 mm), and necrotic areas (CT <30 HU). For multifocal cases, each tumor ≥1 cm received a separate annotation. Initial independent segmentations were compared using Dice coefficients, and discrepancies (DSC <0.7) were adjudicated by a senior radiologist. Consensus VOIs were generated through discussions. The uAI platform adheres to the Imaging Biomarker Standardization Initiative (IBSI) guidelines and follows the Clinical, Laboratory, and Experimental Analysis Report (CLEAR) framework.

Feature extraction was performed using the uAI Research Portal’s radiomics module, incorporating first-order statistics features, shape features, and gray-level-related features (such as gray-level co-occurrence matrix). Features were extracted from both raw images and those processed with various filters, such as box mean, Additive Gaussian Noise, and Binomial Blur Image.

### Construction and validation of radiomics models

The radiomic features were selected sequentially to ensure robustness. First, correlation analysis and preliminary univariate screening were performed to reduce the dimensionality and remove redundant features. This process reduced the number of features from 2,286 to 593 for PD-L1 prediction and 482 for VETC prediction. Subsequently, univariate logistic regression with a stricter threshold (P-value <0.05) was applied to identify the most significant features, resulting in 64 retained features for PD-L1 and 30 for VETC. Finally, the LASSO regression model with 10-fold cross-validation was used to select the most predictive features, yielding seven features for PD-L1 and 10 for VETC. A radiomics score (Radscore) was calculated using the coefficients of the selected features.

Based on the Radscore, a radiomic model was constructed using machine learning algorithms such as AdaBoost (AB), Bagging Decision Tree (BDT), Decision Tree (DT), Logistic Regression (LR), Random Forest (RF), Support Vector Machine (SVM), and eXtreme Gradient Boosting (XGBOOST). Receiver Operating Characteristic (ROC) curves were plotted. The area under the curve (AUC) was used to evaluate the model’s predictive accuracy for PD-L1 and VETC. Decision Curve Analysis (DCA) was used to calculate the net benefit for patients at various threshold probabilities, assessing the clinical utility of each model.

### Statistical analysis

Statistical analyses were conducted using SPSS 26 and R software 4.4.1. Normally distributed continuous variables are described as mean ± SD; non-normally distributed continuous variables are described as median and interquartile range (IQR), and categorical variables are expressed as numbers and percentages. Continuous clinical variables were analyzed using the Student’s t-test or Mann–Whitney U test, and categorical variables were analyzed using Pearson’s chi-square test. Statistical significance was set at p-value <0.05. The Wilcoxon test was used to compare the evaluation efficacy of Radscore between the training and validation groups for PD-L1 and VETC expression. The Brier score and Hosmer–Lemeshow test were used to assess model fit, with p >0.05 indicating a good fit. The DeLong test was used to evaluate the sensitivity, specificity, and AUC of the ROC curves to assess the effectiveness of the classification system.

## Results

### Patient characteristics

This study included 162 patients (male, n = 139; mean age 53.82 ± 12.29), among whom 72 (44.4%) were PD-L1 positive and 84 (51.9%) were VETC positive. Patients were randomly allocated in an 8:2 ratio to the training set (PD-L1+, n = 59, 45.4%; VETC+, n = 68, 52.3%) and test set (PD-L1+, n = 13, 40.6%; VETC+, n = 14, 43.8%). There were no significant differences in the clinical characteristics between the training and test sets (**Table 1**).

**Table 1:** Characteristic baseline of patients.

Variables	Category	Total set (n = 162)	Training set (n = 130)	Test set (n = 32)	P value
Age (years)		53.82 ± 11.74	53.94 ± 12.29	53.36 ± 9.561	0.828
Sex (%)					1
	Male	139 (85.8)	112 (86.2)	27 (84.4)	
	Female	23 (14.2)	18 (13.8)	5 (15.6)	
PD-L1 (%)					0.607
	Negative	90 (55.6)	71 (54.6)	19 (59.4)	
	Positive	72 (44.4)	59 (45.4)	13 (40.6)	
VETC (%)					0.47
	Negative	78 (48.1)	62 (47.7)	18 (56.2)	
	Positive	84 (51.9)	68 (52.3)	14 (43.8)	
Child–Pugh grade (%)					0.775
	A	151 (93.2)	122 (93.4)	29 (90.6)	
	B	11 (6.8)	8 (6.6)	3 (9.4)	
Tumor number (%)					0.63
	1	104 (64.2)	82 (63.1)	22 (68.8)	
	≥2	58 (35.8)	48 (36.9)	10 (31.3)	
Main tumor diameter (mm)		65 (39,94.5)	65 (38.75,95.25)	64 (40.5,88.5)	0.961
Tumor metastasis (%)					0.252
	No	129 (79.6)	101 (77.7)	28 (87.5)	
	Yes	33 (20.4)	29 (22.3)	4 (12.5)	
Venous invasion (%)					0.875
	No	121 (74.7)	97 (74.6)	24 (75)	
	Yes	41 (25.3)	33 (25.4)	8 (25)	
Liver cirrhosis (%)					0.912
	No	112 (69.1)	90 (69.2)	22 (68.8)	
	Yes	50 (30.9)	40 (30.8)	10 (31.3)	
Hepatitis B (%)					0.57
	No	22 (13.6)	19 (14.6)	3 (9.4)	
	Yes	140 (86.4)	111 (85.4)	29 (90.6)	
AFP		251.8 (9.12,3352)	180.75 (9.2475,2996.25)	323.1(6.815,55312.5)	0.62
AST		43 (31,64)	43 (31,64.5)	45 (30,66.5)	0.99
ALT		36 (23,61)	35 (22,61.25)	38 (28.5,62.5)	0.385
Total bilirubin		14.9 (11.1,19.9)	14.45 (11.075,19.75)	15.4 (11.5,21.95)	0.577
GGT		77 (40,142)	80 (40,142)	67 (36,142)	0.538
Albumin		40.1 (36.5,43.7)	40.8 (36.75,43.7)	39.2 (34.3,44.5)	0.708
PT		12.8 (12.2,13.7)	12.95 (12.2,13.85)	12.6 (11.65,13.2)	0.092
Hepatic encephalopathy (%)					1
	No	162 (100)	130 (100)	32(100)	
	Yes	0 (0)	0 (0)	0(0)	
Ascites (%)					1
	No	154 (95.1)	123 (94.6)	31(96.9)	
	Yes	8 (4.9)	7 (5.4)	1(3.1)	

A total of 2,286 features were extracted and selected using correlation coefficients, logistic regression, and LASSO algorithms. Ultimately, we identified seven features strongly associated with PD-L1 and 10 features closely related to VETC. Specifically, the seven PD-L1-related features and five of the 10 VETC-related features showed positive correlations. The Radscore was calculated and used to construct the model. The details of Radscore are provided in **Appendix 1**.

**Figure 2:**
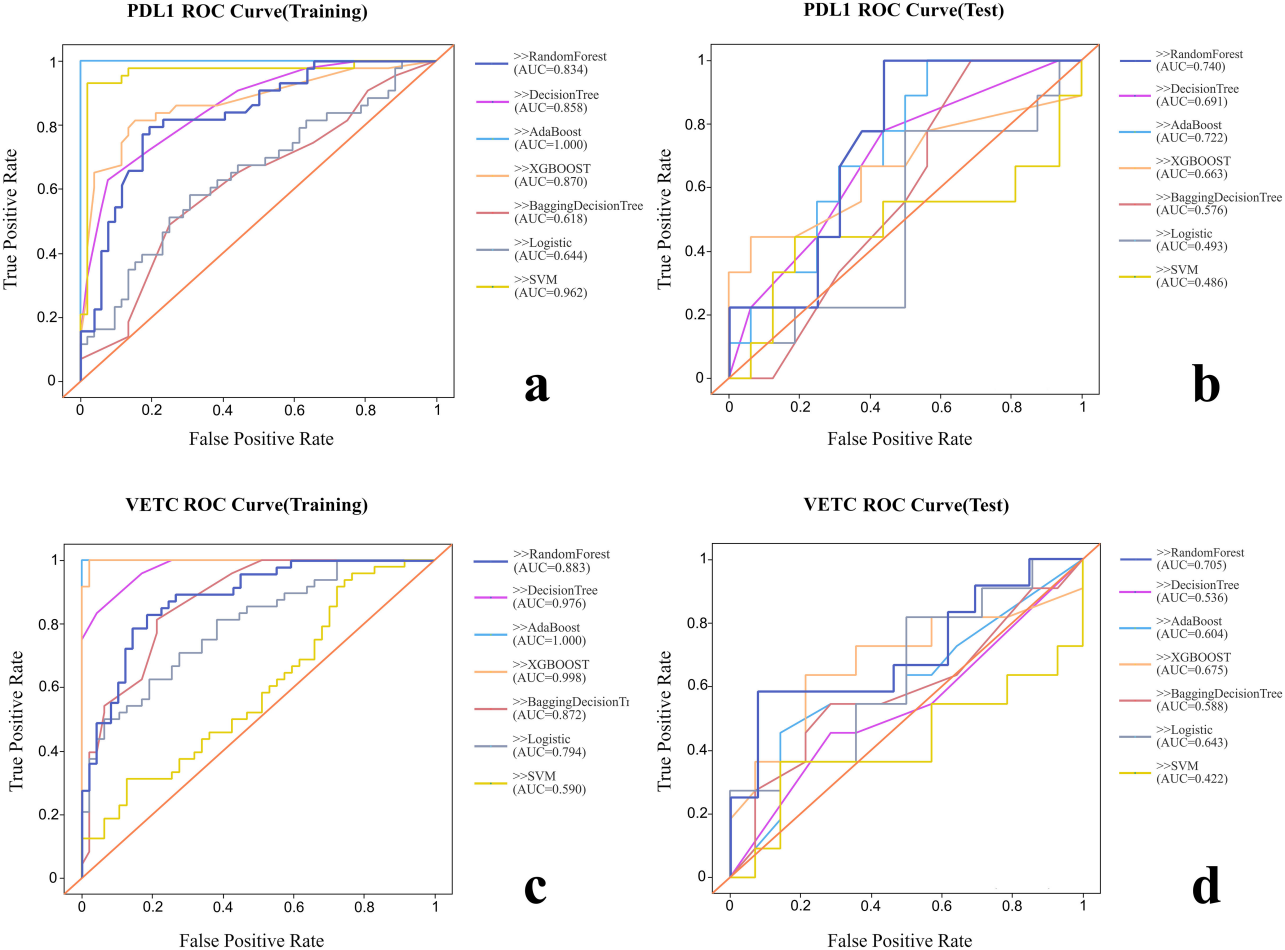
Comparison of the ROC curves of each radiomic model. **(A)** ROC curve of training group for PD-L1. **(B)** ROC curve of test group for PD-L1. **(C)** ROC curve of training group for VETC. **(D)** ROC curve of test group for VETC.

**Figure 3:**
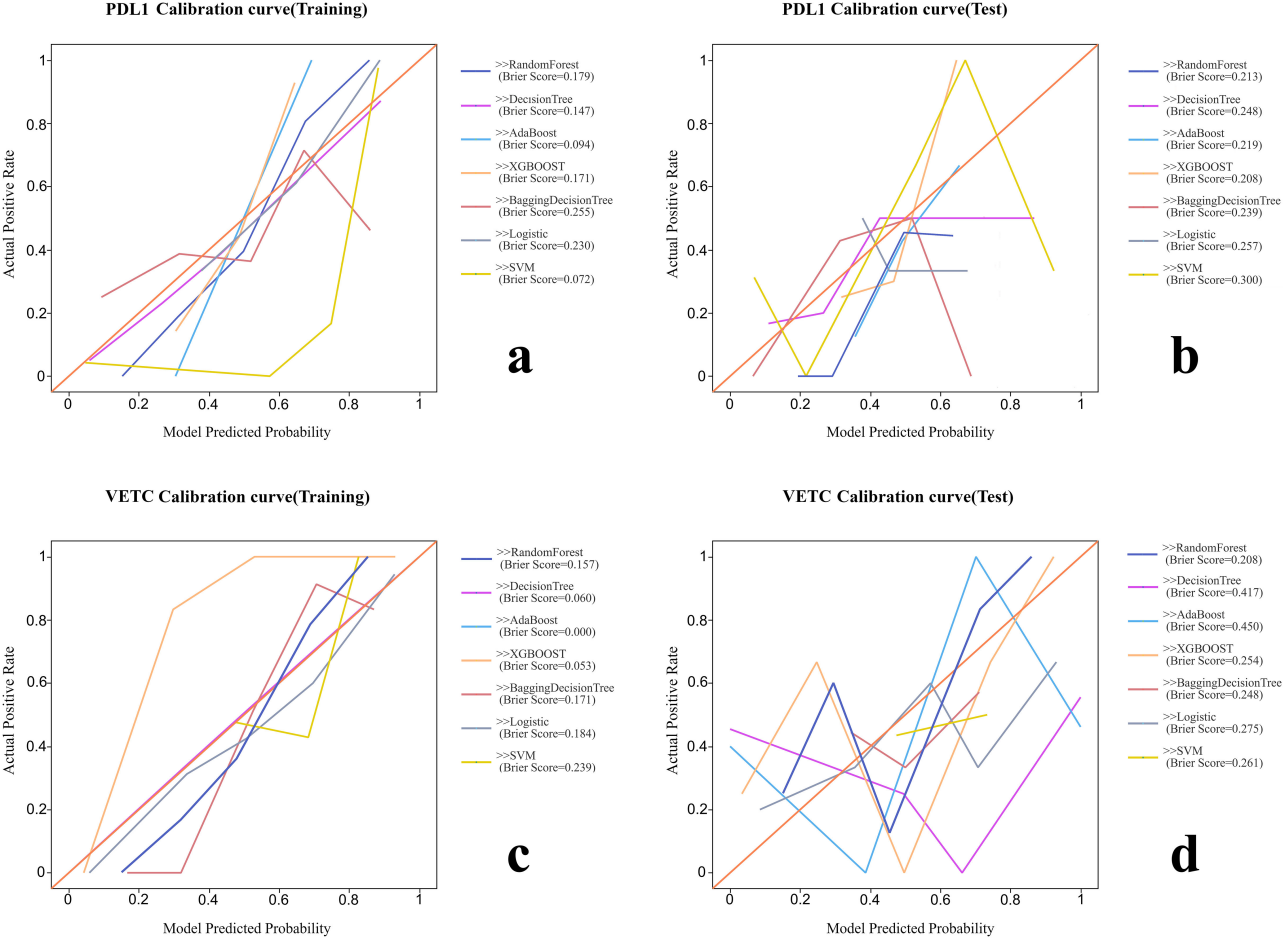
The calibration curves and Brier scores of each radiomic model. **(A)** Calibration curve of training group for PD-L1. **(B)** Calibration curve of test group for PD-L1. **(C)** Calibration curve of training group for VETC. **(D)** Calibration curve of test group for VETC.

**Figure 4:**
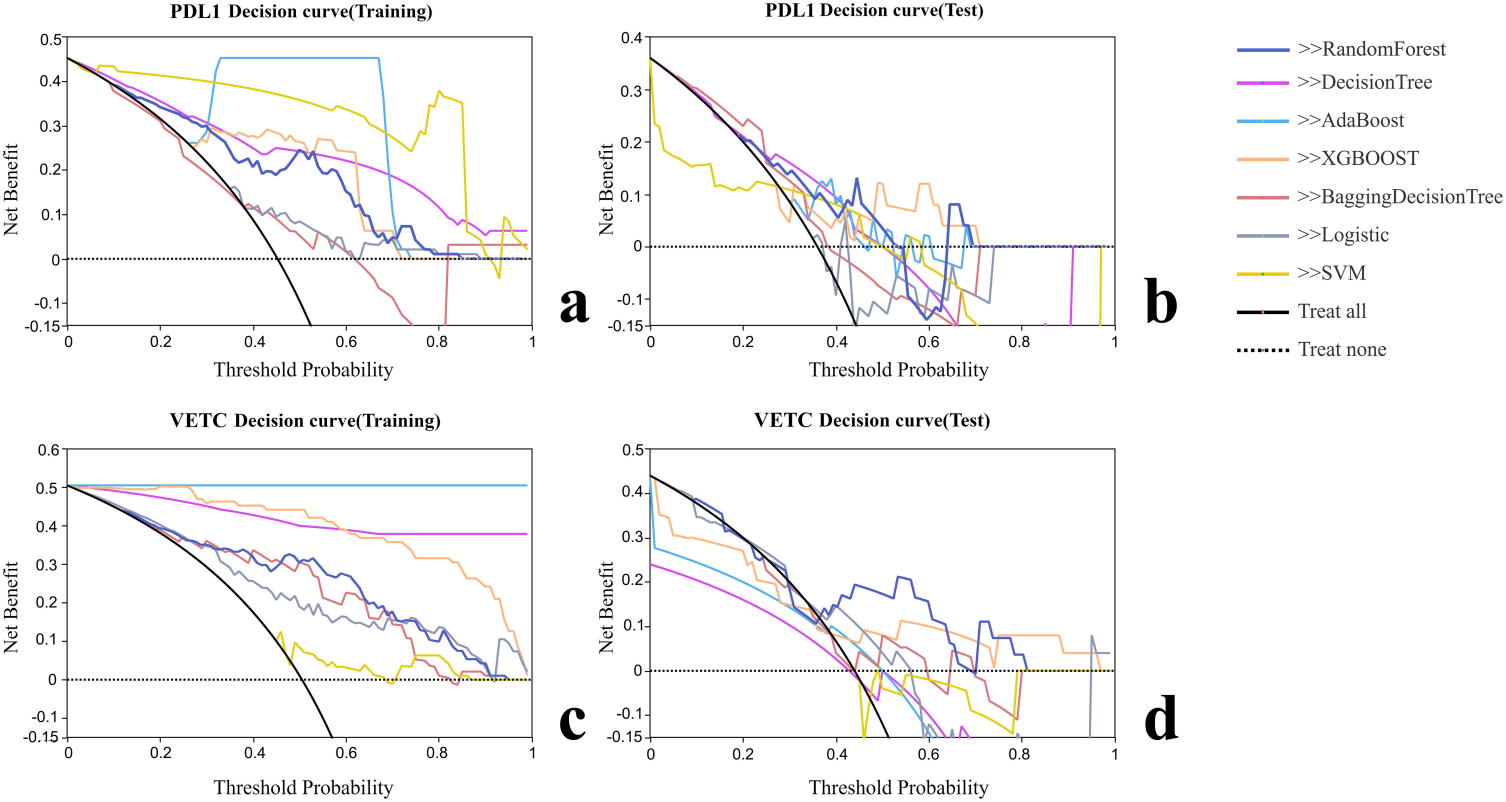
Decision curve analysis of each radiomic model. **(A)** Decision curve analysis of training group for PD-L1. **(B)** Decision curve analysis of test group for PD-L1. **(C)** Decision curve analysis of training group for VETC. **(D)** Decision curve analysis of test group for VETC.

Various radiomic models, including AB, BDT, DT, LR, RF, SVM, and XGBOOST, were used to predict PD-L1 and VETC, and the performance of each model was evaluated (**Table 2**, **Figure 2**). Among them, the RF model demonstrated the best performance, with an AUC of 0.834 (95% CI: 0.752–0.915) for PD-L1 prediction in the training set and 0.740 (95% CI: 0.541–0.939) in the test set. For VETC prediction, RF achieved an AUC of 0.883 (95% CI: 0.818–0.949) in the training set and 0.705 (95% CI: 0.488–0.922) in the test set. To assess the calibration and usefulness of the models, calibration curves and decision curve analyses (DCA) were calculated, and the results are shown in **Figures 3**, **4**. The Hosmer–Lemeshow test (P >0.05) and Brier Score indicated a good model fit for the RF model.

**Table 2:** Performance evaluation of radiomics models in predicting PD-L1 and VETC expression.

Biomarker	Model	Training set					Test set				
		AUC (95%CI)	Sen.	Spe.	Acc.	Pre.	AUC (95%CI)	Sen.	Spe.	Acc.	Pre.
PD-L1	RF	0.834 (0.752–0.915)	0.818	0.731	0.771	0.72	0.74 (0.541–0.939)	0.889	0.562	0.68	0.533
	AB	1 (1–1)	1	1	1	1	0.722 (0.52–0.924)	0.556	0.688	0.64	0.5
	BDT	0.618 (0.504–0.732)	0.651	0.558	0.6	0.549	0.576 (0.352–0.801)	0.222	0.75	0.56	0.333
	DT	0.858 (0.786–0.931)	0.628	0.923	0.789	0.871	0.691 (0.474–0.908)	0.444	0.75	0.64	0.5
	LR	0.644 (0.532–0.757)	0.512	0.731	0.632	0.611	0.493 (0.244–0.742)	0.222	0.688	0.52	0.286
	SVM	0.962 (0.917–1)	0.953	0.865	0.905	0.854	0.486 (0.203–0.769)	0.333	0.812	0.64	0.5
	XGBOOST	0.834 (0.752–0.915)	0.818	0.731	0.771	0.72	0.74 (0.541–0.939)	0.889	0.562	0.68	0.533
VETC	RF	0.883 (0.818–0.949)	0.787	0.837	0.812	0.822	0.705 (0.488–0.922)	0.583	0.846	0.72	0.778
	AB	1 (1–1)	1	1	1	1	0.604 (0.369–0.839)	0.636	0.5	0.56	0.5
	BDT	0.872 (0.803–0.942)	0.812	0.787	0.8	0.796	0.588 (0.346–0.829)	0.545	0.714	0.64	0.6
	DT	0.976 (0.955–0.997)	0.833	0.957	0.895	0.952	0.536 (0.309–0.763)	0.455	0.643	0.56	0.5
	LR	0.794 (0.706–0.882)	0.75	0.617	0.684	0.667	0.643 (0.415–0.87)	0.818	0.429	0.6	0.529
	SVM	0.59 (0.475–0.705)	0.292	0.872	0.579	0.7	0.422 (0.168–0.676)	0.182	0.857	0.56	0.5
	XGBOOST	0.998 (0.994–1)	0.875	1	0.937	1	0.675 (0.44–0.91)	0.364	0.857	0.64	0.667

AB, AdaBoost; BDT, Bagging Decision Tree; DT, Decision Tree; LR, Logistic Regression; RF, Random Forest; SVM, Support Vector Machine; XGBOOST, eXtreme Gradient Boosting.

## Discussion

In this study, we established an imaging radiomics model based on enhanced abdominal CT scans to predict the expression of PD-L1 and VETC in patients with HCC. This model aims to provide noninvasive biomarkers for therapeutic decision-making in patients without available histopathological data. The results demonstrate that the RF model achieved high diagnostic efficacy in identifying PD-L1 and VETC expression.

The AUC for PD-L1 prediction in the test set was 0.740 (95% CI: 0.541–0.939), indicating a moderate discriminative ability. Although it did not reach the ideal threshold (AUC ≥0.8), its noninvasive nature can provide preliminary risk stratification for patients who cannot undergo biopsy, such as prioritizing high-risk patients for immune checkpoint inhibitor treatment. The performance decline in the test set (e.g., VETC AUC dropping from 0.883 to 0.705) may be attributed to the insufficient sample size (n = 32) or differences in feature distribution between the training and test sets. Future validation with larger samples and multicenter data is needed to assess the model’s stability.

Radiomics offers significant predictive advantages owing to its noninvasive nature, enabling the analysis of medical images without invasive procedures. It facilitates high-dimensional data analysis by extracting quantitative features that provide insights into tumor morphology and texture, surpassing the capabilities of traditional imaging methods (14). This approach supports personalized medicine by identifying specific pathological characteristics for tailored treatment plans in patients with HCC. Feature selection was conducted using correlation coefficients, univariate logistic regression, and Lasso regression to enhance predictive capability. The RF model demonstrated good calibration and fit in both the training and testing groups, indicating its effectiveness in predicting PD-L1 and VETC expression, which may be attributed to the ability of its ensemble learning mechanism to capture nonlinear relationships in high-dimensional features. We acknowledge the performance gap observed between the training and test sets, particularly for VETC prediction (AUC decrease from 0.883 to 0.705). This pattern is indicative of potential overfitting, a common challenge in radiomics studies, characterized by high dimensionality relative to the sample size. Notably, compared to the Random Forest model, other algorithms, such as AdaBoost, achieved an AUC of 1.000 in the training set but suffered more severe performance declines in the test set, highlighting their sensitivity to noise in high-dimensional data. This observation reinforces our rationale for selecting the Random Forest classifier, which utilizes ensemble methods to better manage variance. The observed performance fluctuations are likely attributable to several factors: the high feature-to-sample ratio, the inherent heterogeneity of HCC not fully captured in the limited test partition (n = 32), and the lack of nested cross-validation. Although we employed LASSO feature selection to mitigate these issues, future validation in larger, multicenter cohorts is essential to confirm model generalization. Moreover, it aims to provide noninvasive biomarkers for patients without tissue pathology data, thereby assisting in clinical decision-making and improving patient outcomes.

Consistent with studies by Dong et al. (15), Zhang et al. (16), and Xie et al. (17), our radiomics model leverages imaging data to predict the pathological presentations of HCC preoperatively with notable efficacy and reproducibility. This confirms the correlation between radiographic images and the expression of tumor pathology. Recent studies have highlighted the potential of radiomics in decoding the tumor immune microenvironment of HCC. For instance, radiomic signatures have been shown to correlate with the density of tertiary lymphoid structures and the infiltration of CD8+ T cells, offering noninvasive insights into the immune landscape (18, 19). Our findings align with this emerging evidence, suggesting that CT-based features capture subtle textural patterns associated with immune-related biomarkers, such as PD-L1. Compared to prior research, our RF model integrates predictions of PD-L1 and VETC, offering a comprehensive assessment that can more thoroughly evaluate and predict the disease state of patients, thereby guiding therapeutic decision-making.

High PD-L1 expression is closely associated with the immune evasion mechanisms of tumors and the formation of the tumor microenvironment, significantly correlating with patient prognosis (20). VETC has been proven to be particularly related to extrahepatic metastasis, especially to the lungs, compared to other distant metastases (21). The expression of PD-L1 and VETC has been identified as an independent predictor of poor prognosis in patients with HCC, indicating a higher likelihood of tumor recurrence and metastasis (22–25). Assessing the expression status of PD-L1 and VETC can guide clinical decision-making and personalized treatment for HCC patients (26). For instance, the level of PD-L1 expression can direct the selection of ICI therapy protocols in liver cancer patients, as suggested by the KEYNOTE-224 study, in which pembrolizumab showed superior efficacy in patients with higher levels of PD-L1 expression (27). Additionally, VETC-positive cases are more sensitive to TACE, HAIC, and targeted therapies, deriving more treatment benefits than VETC-negative cases (9, 28–30). Our constructed radiomics model exhibited good sensitivity and specificity in predicting the expression of PD-L1 and VETC, holding potential clinical application value to aid in the formulation of clinical treatment plans and prognosis evaluation.

While our model demonstrated promising results, the retrospective single-center design and relatively limited sample size, particularly in the independent test set (n = 32), represent primary limitations. The observed performance fluctuations between the training and test cohorts may be partially attributed to the small sample size and potential distribution shifts in the high-dimensional features. Consequently, the current results should be interpreted as being preliminary. Future studies utilizing large-scale, multicenter cohorts are essential to validate the model’s generalizability and robustness across diverse populations. Moreover, the study primarily relied on CT imaging data and utilized arterial phase imaging. Incorporating multimodal imaging techniques, such as MRI, and integrating multi-sequence imaging information could provide more comprehensive insights and improve the accuracy of the prediction model in future investigations.

In addition, HCC that concurrently exhibits both VETC and classical vascular structures (VETC±) has a worse prognosis than HCC that solely expresses VETC− or VETC +. Patients with VETC± have shorter survival times and higher recurrence rates. Pathological evidence suggests that in VETC± HCC, liver metastasis is more likely to occur through an epithelial–mesenchymal transition (EMT)-dependent mechanism associated with VETC− rather than via the VETC pathway (31). This variation in expression influences treatment choice and prognosis. In this study, we did not separate the VETC± cases from the VETC+ cases. Future studies should isolate these cases for dedicated radiomic analyses.

Future research should address these limitations and explore the integration of radiological models into clinical decision-making processes to improve treatment outcomes and prognosis in patients with HCC.

## Conclusion

A radiomic model established using random forests (RF) based on enhanced abdominal CT demonstrated the potential to non-invasively predict PD-L1 and VETC expression in HCC patients. This model may provide preliminary information to assist clinicians in guiding clinical decision-making and developing personalized treatment plans, particularly for patients lacking histopathological data, ultimately aimed at improving therapeutic efficacy and prognosis for HCC patients.

## Data Availability

The raw data supporting the conclusions of this article will be made available by the authors, without undue reservation.
