# Supplementary material for: A radiomic model for noninvasive prediction of PD-L1 and VETC expression in hepatocellular carcinoma using enhanced abdominal CT
Source: Front Oncol. 2025 Dec 12;15:1696376. doi: 10.3389/fonc.2025.1696376 (PMC12740864; doi:10.3389/fonc.2025.1696376)
Supplement: Supplementary file 1 [file DataSheet1.docx]

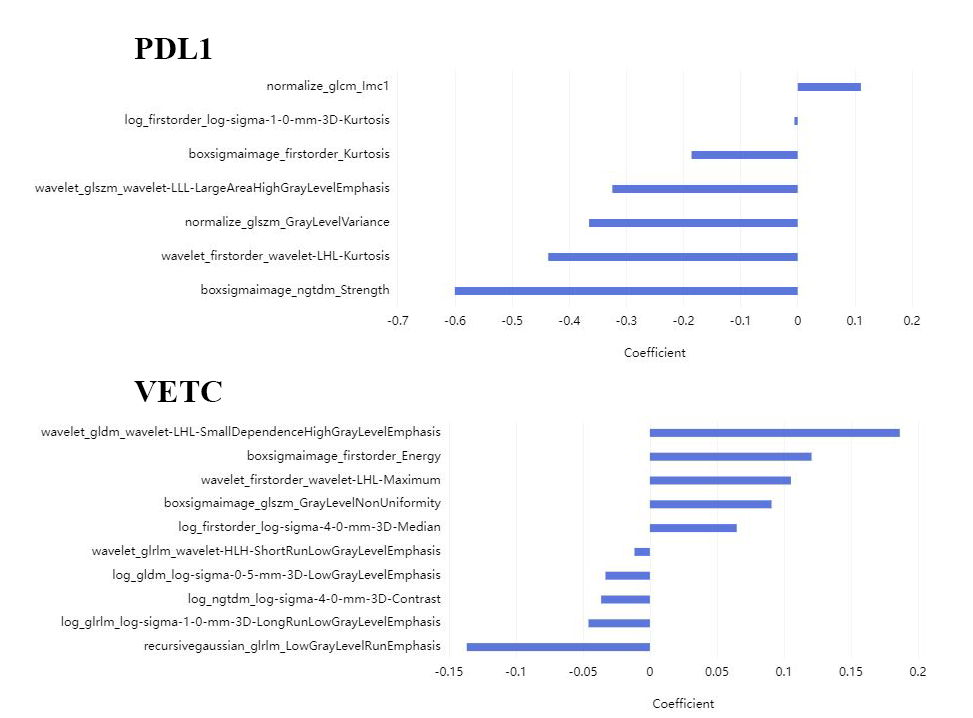


Features strongly associated with PD-L1 and VETC.

PDL1 Radscore =

0.11055924 * normalize_glcm_Imc1

+ -0.005726956 * log_firstorder_log-sigma-1-0-mm-3D-Kurtosis

+ -0.185813025 * boxsigmaimage_firstorder_Kurtosis

+ -0.324425757 * wavelet_glszm_wavelet-LL-LargeAreaHighGrayLevelEmphasis

+ -0.365141183 * normalize_glszm_GrayLevelVariance

+ -0.4365912 * wavelet_firstorder_wavelet-LHL-Kurtosis

+ -0.6000737* boxsigmaimage_ngtdm_Strength

+ 0.7483548

VETC Radscore =

0.186430633 * wavelet_gldm_wavelet-LHL-SmallDependenceHighGrayLevelEmphasis

+ 0.120599955 * boxsigmaimage_firstorder_Energy

+ 0.105206117 * wavelet_firstorder_wavelet-LHL-Maximum

+ 0.09074153 * boxsigmaimage_glszm_GrayLevelNonUniformity

+ 0.0647860542 * log_firstorder_log-sigma-4-0-mm-3D-Median

+ -0.011508585 * wavelet_glrlm_wavelet-HLH-ShortRunLowGrayLevelEmphasis

+ -0.0331391133 * log_gldm_log-sigma-0-5-mm-3D-LowGrayLevelEmphasis

+ -0.03635304 * log_ngtdm_log-sigma-4-0-mm-3D-Contrast

+ -0.04580407 * log_glrlm_log-sigma-1-0-mm-3D-LongRunLowGrayLevelEmphasis

+ -0.136617273* recursivegaussian_glrlm_LowGrayLevelRunEmphasis

+ 0.445031732
